# Validation of the binding stoichiometry between HCN channels and their neuronal regulator TRIP8b by single molecule measurements

**DOI:** 10.3389/fphys.2022.998176

**Published:** 2022-09-26

**Authors:** Andrea Saponaro, Francesca Vallese, Alessandro Porro, Oliver B. Clarke

**Affiliations:** ^1^ Department of Biosciences, University of Milan, Milano, Italy; ^2^ Department of Physiology and Cellular Biophysics, Columbia University, New York, NY, United States; ^3^ Department of Anesthesiology, Columbia University Irving Medical Center, New York, NY, United States; ^4^ Irving Institute for Clinical and Translational Research, Columbia University, New York, NY, United States

**Keywords:** HCN channels, I_h_ current, TRIP8b, cAMP, mass photometry, stoichiometry

## Abstract

Tetratricopeptide repeat–containing Rab8b-interacting (TRIP8b) protein is a brain-specific subunit of Hyperpolarization-activated Cyclic Nucleotide-gated (HCN) channels, a class of voltage-gated channels modulated by cyclic nucleotides. While the interaction between TRIP8b and the cytosolic C terminus of the channel has been structurally described, the HCN:TRIP8b stoichiometry is less characterized. We employed single molecule mass photometry (MP) to image HCN4 particles purified in complex with TRIP8b. Our data show that four TRIP8b subunits are bound to the tetrameric HCN4 particle, confirming a 1:1 stoichiometry.

## Introduction

Hyperpolarization-activated cyclic nucleotide-gated (HCN1–4) channels conduct the h-current (I_h_), which plays a critical role in regulating several neuronal properties, including membrane resting potential, dendritic excitability, and intrinsic rhythmicity (Robinson and Siegelbaum, 2003). HCNs belong to the superfamily of tetrameric voltage-gated K^+^ channels (Kv) and are uniquely regulated by the direct binding of cAMP to their cytoplasmic Cyclic Nucleotide Binding Domain (CNBD). In addition to cAMP, neuronal HCN channels are further modulated by TRIP8b, a brain-specific cytoplasmic subunit, which controls channel trafficking and gating (Santoro et al., 2011). Particularly, TRIP8b, by interacting with the CNBD and thus impairing cAMP binding, antagonizes the facilitatory effect of the cyclic nucleotide on the voltage-dependent gating of HCN channels (Hu et al., 2013).

The structural details of the mechanism of TRIP8b binding to the C terminus of HCN channels are well known (Bankston et al., 2017; Deberg et al., 2015; Hu et al., 2013; Porro et al., 2020; Saponaro et al., 2014). TRIP8b possesses at least two binding sites for HCN channels: 1) a core sequence of 40 amino acid, named TRIP8b_nano_, that binds to the CNBD in a cAMP-dependent manner (Saponaro et al., 2018); 2) the tetratricopepide (TPR) domain of TRIP8b that interacts with the last three amino acids (SNL) of the channel (Bankston et al., 2012). TRIP8b_nano_ sequence is necessary and sufficient to inhibit the cAMP-dependent activation of HCN channels (Porro et al., 2020; Santoro et al., 2011; Saponaro et al., 2018; Hu et al., 2013).

The association stoichiometry between TRIP8b and the HCN tetramer is less characterized. By using single molecule photobleaching method, Bankston and co-workers have shown that HCN2 and EGFP-TRIP8b form a 4:4 complex in the membrane of *Xenopus laevis* oocytes (Bankston et al., 2012). Given that, often times, results obtained with this approach were further disproved by direct biochemical/structural data (Balasuriya et al., 2014; Hou et al., 2012; Ji et al., 2008; Yen et al., 2016), we decided to confirm the HCN:TRIP8b stoichiometry with a different approach.

Here, we present the results of the binding stoichiometry of HCN channels and TRIP8b performed by single molecule mass photometry (MP). Mass Photometry enables mass measurement of single molecules in solution by detecting light scattering as they non-specifically and transiently interact with a glass surface. Binding events change the refractive index at the water/glass interface. This alteration of the local reflectivity can be converted into the molecular mass of the molecule (Young et al., 2018).

By mass-imaging detergent-purified HCN4 - TRIP8b complexes, we detected four TRIP8b molecules bound to a tetrameric HCN4 channel particle. Addition of saturating concentrations of cAMP, which competes with TRIP8b for the binding to HCN channels (Hu et al., 2013), caused the disruption of the complex into the two single elements.

Our results confirm the 4:4 HCN2:TRIP8b stoichiometry previously shown by single molecule photobleaching experiments (Bankston et al., 2012) and provides data on cAMP/TRIP8b competition in purified full length HCN proteins.

## Results

**FIGURE 1:**
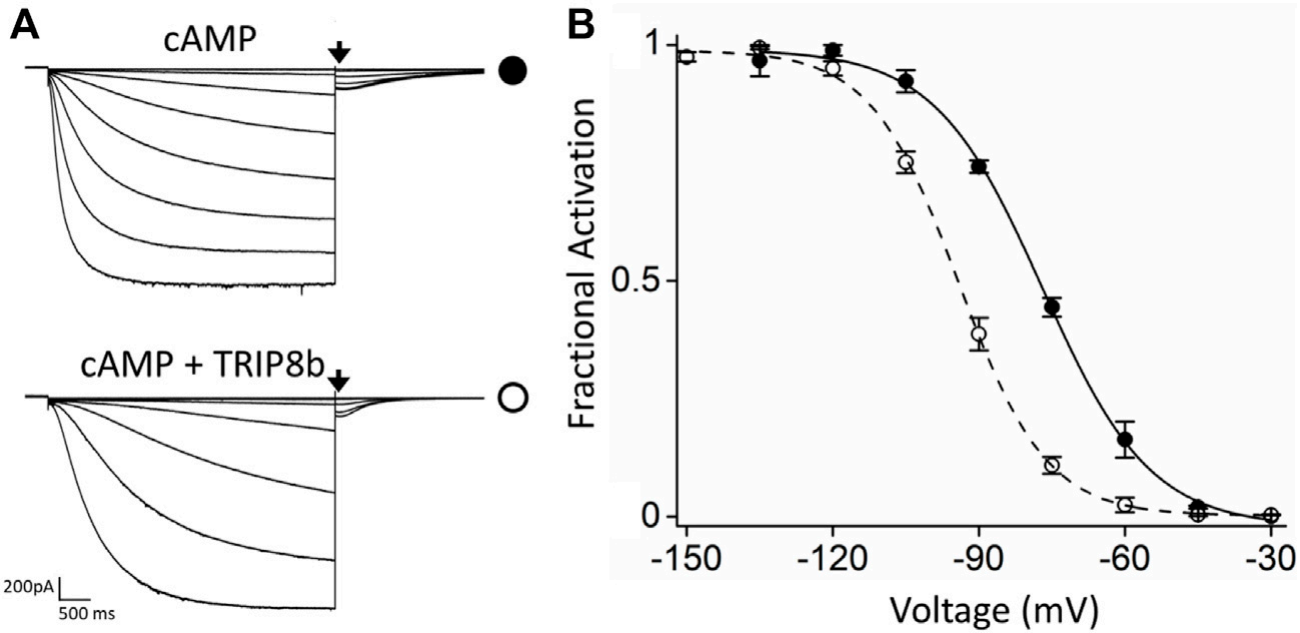
Functional characterization of the inhibitory effect of TRIP8b on the HCN4 construct employed for MP. **(A)** Representative whole-cell current traces of HCN4 channels recorded, with 0.25 µM cAMP in the patch pipette, in HEK293T cells transiently expressing the channel alone (top) or with GFP-TRIP8b (1a) (bottom). Black arrows indicate the current selected for analysis in **(B)**. **(B)** Mean activation curves of HCN4 channels alone (black full circles) or co-expressed with GFP-TRIP8b (1a) (black open circles) with cAMP in the patch pipette obtained from tail currents collected at −40 mV (see arrows in panel **(A)**. Dashed lines indicate Boltzmann fitting to the data (see Materials and methods) from which the half activation potential (V_1/2_) were derived. HCN4 + cAMP = −76.9 ± 0.6 mV; HCN4 + TRIP8b + cAMP = −94.2 ± 1.2 mV. Data are presented as mean ± SEM. Number of cells (N) ≥ 8. The two half activation potentials are statistically different. Statistical analysis performed with t-student test (*p* < 0.001).

For MP studies, HCN4 channels carrying an internal deletion (see Material and Methods for details) (Saponaro et al., 2021a) and EGFP- tagged TRIP8b (1a), one of the most abundant TRIP8b isoform in brain (Santoro et al., 2009), were transiently co-expressed in HEK293F cells by using a HCN4:TRIP8b DNA ratio of 1:1 to do not bias MP results by overexpressing TRIP8b. Electrophysiological analysis performed on HEK293T cells transiently expressing EGPF-TRIP8b (1a) together with the modified HCN4 channels (hereafter indicated as HCN4) using the same 1:1 DNA ratio (Figure 1) showed that TRIP8b fully antagonizes the cAMP effect as previously reported for full length HCN4 channels (Saponaro et al., 2018).

**FIGURE 2:**
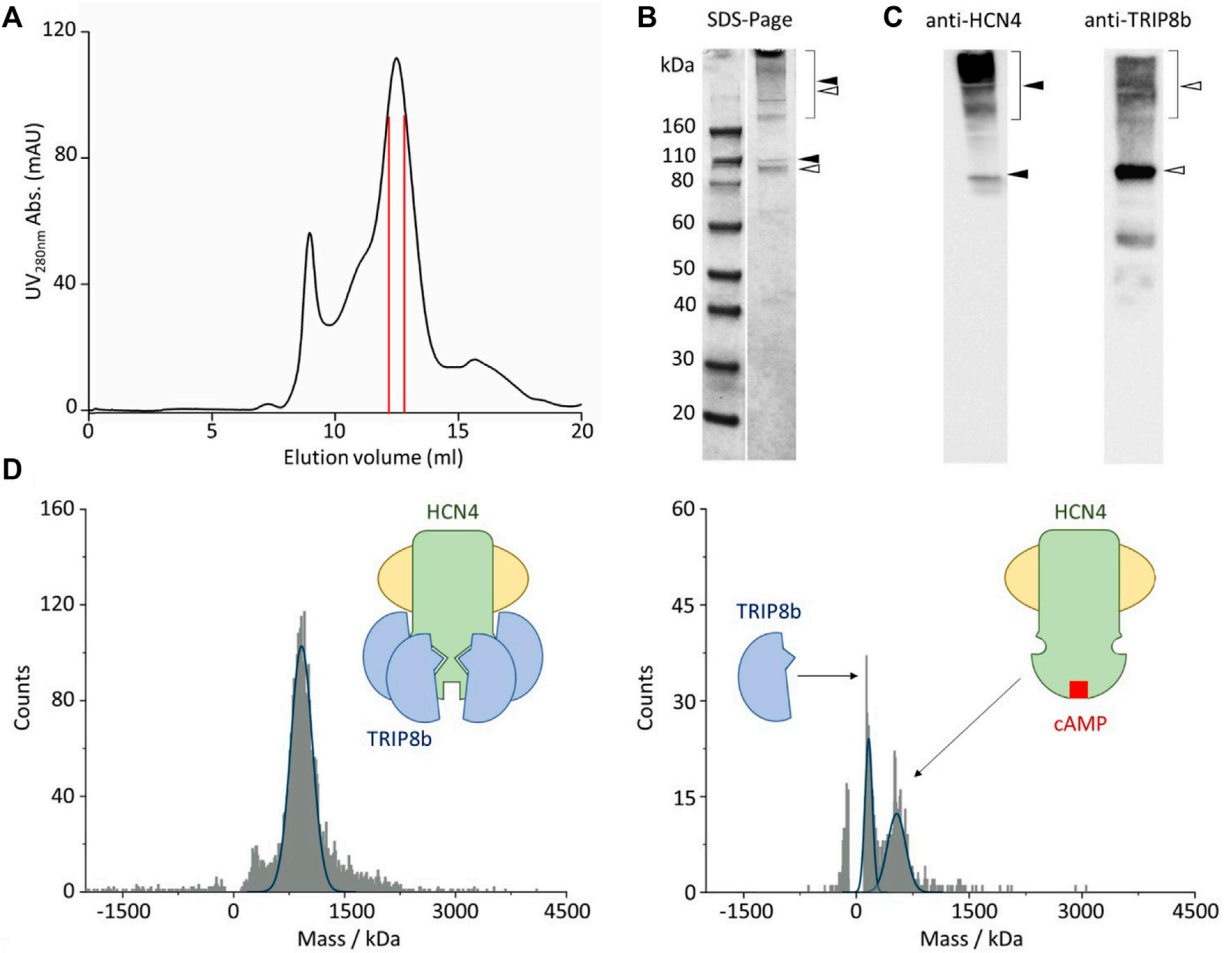
Single molecule mass photometry of HCN4—EGFP-TRIP8b complex. **(A)** SEC of HCN4—EGFP-TRIP8b complex following purification in LMNG/CHS. Peak fractions used for MP are delimited by the red lines. **(B)** SDS-PAGE gel of the pooled SEC fractions of HCN4—EGFP-TRIP8b complex, stained with Coomassie blue. Filled arrows indicate HCN4, while open arrows indicate EGFP-TRIP8b, in their oligomeric (square bracket), and monomeric forms. **(C)** Western blots of the pooled SEC fractions of HCN4—EGFP-TRIP8b complex performed by using anti-TRIP8b and anti-HCN4 antibodies, respectively. Open and filled arrows as in panel **(B)**. Of note that the western blot anti-TRIP8b reveals the presence of a small number of molecules (faint band between 60 and 50 kDa markers) corresponding N terminally degraded TRIP8b. Indeed, they co-purified with HCN4 and thus have retained the HCN binding sites located in their C-terminal half (Santoro et al., 2004; Santoro et al., 2011). **(D)** Mass histogram of binding events for HCN4—EGFP-TRIP8b complex before (left) and after the addition of 2 mM cAMP (right). The blue lines represent the fitting of the data with Gaussian Distribution Function. Left, schematic representation of purified HCN4 (green) embedded into a detergent micelle (yellow) and bound to four TRIP8b molecules (blue). Right, purified HCN4 bound to cAMP (red) has lost TRIP8b.

After membrane solubilization with detergents, the HCN4—TRIP8b complex was affinity purified by using the polyhistidine tag at the N-terminus of EGFP-TRIP8b. The complex was further isolated by size-exclusion chromatography and the co-elution of HCN4 and EGFP-TRIP8b was confirmed by immunoblot (Figures 2A–C). Of note that HCN4 does not completely lose its quaternary structure when run into a denaturing gel (Figures 2B,C, left panel). This was already reported for detergent-purified HCN1 (Lee and MacKinnon, 2017) and HCN4 (Saponaro et al., 2021a; Saponaro et al., 2021b). The same occurs also for HCN4 - TRIP8b complex, although in the latter case the phenomenon is significantly reduced since most of TRIP8b molecules run in their monomeric state (Figures 2B,C, right panel). This is expected as TRIP8b is a soluble protein and thus it is more prone to be denatured and consequently to dissociate from HCN4.

The complex was then imaged with MP. Figure 2D shows the mass distribution histograms of the purified HCN4 - EGFP-TRIP8b complex. Each histogram represents a single molecule binding signal arising from 1-min recording of binding events to the glass surface. HCN4—TRIP8b complex produced a mostly homogeneous mass distribution with a main peak of 935 kDa. The theoretical molecular weight (MW) of the complex with 4:4 stoichiometry is 768 kDa since the expected MW for the monomeric HCN4 is 98.4 kDa and that of EGFP-TRIP8b is 93.6 kDa. Given the absence of protein contaminants (Figures 2B,C), The about 167 kDa difference between the calculated and the theoretical MW of HCN4 - EGFP-TRIP8b complex may be ascribed to the mass of the LMNG-CHS detergent micelle surrounding HCN4. It is worth noting that the micelles are dynamic structures in equilibrium with the surrounding environment. This introduces a certain degree of variability in the mass of a detergent-purified membrane protein.

We further tested the complex by adding an excess of cAMP (2 mM) that should disrupt the interaction, leading to the appearance of two peaks: 1) the homotetrameric HCN4 channel embedded into the detergent micelle (expected mass of about 500kDa); 2) the monomeric EGFP-TRIP8b (expected mass of 93.6 kDa). In line with our prediction, in the presence of cAMP, the 935 kDa peak disappeared and was substituted by two peaks of 498 and 100 kDa respectively (Figure 2D). Of note that the binding events recorded from the sample with cAMP (Figure 2D, right panel) are less than the ones recorded from the sample without the ligand (Figure 2D, left panel). This is due to the dilution of HCN4—EGFP-TRIP8b complex caused by the addition of 2 mM cAMP.

## Discussion

Here we present the measurement of the association stoichiometry of HCN:TRIP8b complex based on a highly accurate determination of the mass of their complex. Such high resolution was achieved by mass photometry (MP). Currently used methods for assessing the mass of macromolecules are solution-based ensemble techniques, with limited mass accuracy and resolution. Instead, MP determines the mass of biomolecules with single molecule sensitivity. The latter feature provides the powerful advantage of an unprecedented resolution/precision, and thus mass accuracy (∼2% mass error) (Young et al., 2018). Therefore, MP allowed us to conclusively establish that TRIP8b forms a tetrameric assembly with HCN4 channels. This finding validates the 4:4 stoichiometry of HCN2:TRIP8b previously determined by Bankston et al. (2012) with fluorescence bleaching.

The finding that TRIP8b shows 4:4 stoichiometry with HCN2 and HCN4 allows to speculate that this will be the case for HCN1 as well, given the high degree of conservation between the three isotypes.

### What is the significance of the 4:4 stoichiometry?

HCN channels play a crucial role in regulating dendritic excitability by filtering excitatory inputs (Magee, 1999; Williams and Stuart, 2000; Tsay et al., 2007; George et al., 2009). Therefore, HCN channels need to be finely controlled, in particular in response to the complexity of cAMP dynamics. TRIP8b is expressed in the brain to this end. In hippocampal CA1 pyramidal neurons, for instance, the sensitivity of I_h_ current to cAMP decreases symmetrically along the dorsoventral axis in well agreement with the dorsoventral gradient of TRIP8b (Marcelin, 2012). Given the pronounced cooperativity of HCN channels in cAMP binding and activation (Ulens and Siegelbaum, 2003; Kusch et al., 2010; Lolicato et al., 2011; Wu et al., 2011; Chow et al., 2012; Kusch et al., 2012; Thon et al., 2015) it is likely to conclude that the obligate saturation of TRIP8b molecules to the four subunits forming the channel appears to be the fastest way possible to strictly and finely control the response of I_h_ to cAMP fluctuations.

A major advantage of MP measurement is the small amount and the low concentration of the protein sample employed (1 μL at 200 nM concentration). This is extremely relevant for eukaryotic membrane proteins as they are difficult to purify in large amounts. In the case of HCN proteins, such limitation has long restricted the biochemical studies of HCN-TRIP8b interaction to their isolated soluble domains (Bankston et al., 2012; Bankston et al., 2017; Deberg et al., 2015; Porro et al., 2020; Saponaro et al., 2014; Saponaro et al., 2018) and thus prevented the study of the complex in full length proteins.

Our study further confirms that the interaction of the two partners can be disrupted by increasing amount of cAMP (Bankston et al., 2017; Hu et al., 2013; Saponaro et al., 2014). Such a competitive action of cAMP on TRIP8b was so far never reproduced in isolated full length HCN proteins. This is particularly relevant in the light of recent functional studies (Gross et al., 2018; Porro et al., 2019), based on the cryo-EM structures of HCN1 (Lee and MacKinnon, 2017), which assigned a key role in the propagation of the cAMP-pathway to an unknown cytoplasmic N terminal domain (HCND) that wedges in between the voltage sensor module and C terminal cytoplasmic regulatory region. This implies that the HCND, as well as other intracellular modules, may contribute to regulate the association between HCN and TRIP8b, and that the interaction between the two proteins is considerably more complex than the mechanism so far described.

## Methods

### Constructs

The cDNA encoding full-length mouse TRIP8b (splice variant 1a4) was cloned into a modified pEG BacMam vector (Goehring et al., 2014) (hereafter pEGA). The cDNA encoding rabbit HCN4 carrying an internal deletion (from residues 783 to 1064, hereafter HCN4) was cloned into pCI vector (Promega Corporation). Is it worth noting that the internal deletion eliminates a poorly conserved region in the C-terminal portion of the HCN channel protein, but preserves the extreme C-terminal SNL tripeptide sequence responsible for binding to TRIP8b (Santoro et al., 2004; Santoro et al., 2011).

### Electrophysiology

HEK293-T cells (ATCC) were cultured, transiently transfected, and measured by patch-clamp technique using a ePatch amplifier (Elements srl) as described in Porro et al. (2019), Porro et al. (2020), Saponaro et al. (2018).

#### Protein complex expression and purification

Freestyle HEK293-F cell cultures (Thermo Fisher) were transiently co-transfected with pCI: HCN4ΔC (0.75 μg per ml) and pEGA: TRIP8b (0.75 µg per ml) according to the procedure detailed in Saponaro et al. (2021a).

Membrane isolation and HCN4—TRIP8b complex purification were pursed according to the protocols details in Saponaro et al. (2021a), with the following modification: HCN4—TRIP8b complex eluted form Ni^2+^-NTA resin (Qiagen) was loaded on a Superose 6 increase 10/300 GL SEC column (GE Healthcare Life Sciences) pre-equilibrated with buffer containing 200 mM NaCl, 20 mM HEPES pH 7.0 and detergent (LMNG-CHS) at the concentration of 0.002% (w/v). Peak fractions (see Figure 2A) were used for mass photometry. For the protein complex analyzed in the presence of cAMP, 2 mM of ligand was added to the sample prior mass photometry.

### SDS-page denaturing gel and western blots

Protein samples were resolved by precast 4–12% SDS-PAGE gels (Thermo Fisher Scientific) and either stained with Coomassie Brilliant Blue R (Merck) or transferred to PVDF membranes (Thermo Fisher Scientific) for Western blotting. Primary antibody dilutions were as follows: anti-HCN4 (rabbit polyclonal, Alomone) 1:1000; anti-TRIP8b (mouse monoclonal, NeuroMab) 1:1000.

Anti-mouse alkaline phosphatase conjugated antibody (Merck) or anti-rabbit alkaline phosphatase conjugated antibody (Merck) diluted 1:1000 were used as secondary antibodies. The protein bands were visualized using SIGMAFAST BCIP^®^/NBT reagent (Merck).

### Mass photometry (MP, iSCAMS)

Mass photometry experiments were performed with a Refeyn OneMP (Refeyn Ltd.). Data acquisition was performed using AcquireMP (Refeyn Ltd. 172 v2.3). Samples were evaluated with microscope coverslips (70 × 26 174 mm). The coverslips were washed with ddH2O and isopropanol. A silicone template was placed on top of the coverslip to form reaction chambers immediately prior to measurement. The instrument was calibrated using NativeMark Protein Standard (Thermo Fisher). 10 μL of fresh room temperature buffer was pipetted into a well, the focal position was identified and locked. For each acquisition 1 μL of the protein (at a concentration of 200 nM) was added to the well and thoroughly mixed. MP signals were recorded for 60 s to allow detection of at least 2 × 103 individual protein molecules. The data were analyzed using the Refeyn AcquireMP 2.3.0 software.

## Data Availability

The raw data supporting the conclusions of this article will be made available by the authors, without undue reservation.
