# Corrigendum: Validation of the binding stoichiometry between HCN channels and their neuronal regulator TRIP8b by single molecule measurements

**DOI:** 10.3389/fphys.2023.1211400

**Published:** 2023-05-03

**Authors:** Andrea Saponaro, Francesca Vallese, Alessandro Porro, Oliver B. Clarke

**Affiliations:** ^1^ Department of Biosciences, University of Milan, Milano, Italy; ^2^ Department of Physiology and Cellular Biophysics, Columbia University, New York, NY, United States; ^3^ Department of Anesthesiology, Columbia University Irving Medical Center, New York, NY, United States; ^4^ Irving Institute for Clinical and Translational Research, Columbia University, New York, NY, United States

**Keywords:** HCN channels, Ihcurrent, TRIP8b, cAMP, mass photometry, stoichiometry

In the published article, there was an error in the **Funding** statement. Two relevant sources of funding were omitted. The correct **Funding** statement appears below.

The authors apologize for this error and state that this does not change the scientific conclusions of the article in any way. The original article has been updated.

